# Root Morphology, Allometric Relations and Rhizosheath of Ancient and Modern Tetraploid Wheats (*Triticum durum* Desf.) in Response to Inoculation with *Trichoderma harzianum* T-22

**DOI:** 10.3390/plants11020159

**Published:** 2022-01-07

**Authors:** Rocco Bochicchio, Rosanna Labella, Antonella Vitti, Maria Nuzzaci, Giuseppina Logozzo, Mariana Amato

**Affiliations:** 1 School of Agriculture, Forestry, Food and Environmental Sciences, University of Basilicata, 85100 Potenza, Italy; rosannalabella@yahoo.it (R.L.); avitti@unisa.it (A.V.); maria.nuzzaci@unibas.it (M.N.); giuseppina.logozzo@unibas.it (G.L.); 2 Department of Pharmacy, University of Salerno, 84100 Salerno, Italy

**Keywords:** root length, root/shoot ratio, specific root length, Saragolle Lucana, seed coating

## Abstract

Early root traits and allometrics of wheat are important for competition and use of resources. They are under-utilized in research and un-explored in many ancient wheats. This is especially true for the rhizosheath emerging from root-soil interactions. We investigated root morphology, root/shoot relations and the amount of rhizosheath of four tetrapoid wheat seedlings (30 days after emergence): the italian landrace Saragolle Lucana and modern varieties Creso, Simeto and Ciclope, and tested the hypothesis that inoculation with *Trichoderma harzianum* T-22 (T-22) enhances rhizosheath formation and affects wheat varieties differently. Overall growth of non-inoculated plants showed different patterns in wheat varieties, with Saragolle and Ciclope at the two extremes: Saragolle invests in shoot rather than root mass, and in the occupation of space with highest (*p* < 0.05) shoot height to the uppermost internode (5.02 cm) and length-to-mass shoot (97.8 cm g^−1^) and root (more than 140 m g^−1^) ratios. This may be interpreted as maximizing competition for light but also as a compensation for low shoot efficiency due to the lowest (*p* < 0.05) recorded values of optically-measured chlorophyll content index (22.8). Ciclope invests in biomass with highest shoot (0.06 g) and root (0.04 g) mass and a thicker root system (average diameter 0.34 mm vs. 0.29 in Saragolle) as well as a highest root/shoot ratio (0.95 g g^−1^ vs. 0.54 in Saragolle). Rhizosheath mass ranged between 22.14 times that of shoot mass in Ciclope and 43.40 in Saragolle (different for *p* < 0.05). Inoculation with *Trichoderma* increased the amount of rhizosheath from 9.4% in Ciclope to 36.1% in Simeto and modified root architecture in this variety more than in others. Ours are the first data on roots and seedling shoot traits of Saragolle Lucana and of *Trichoderma* inoculation effects on rhizosheath. This opens to new unreported interpretations of effects of *Trichoderma* inoculation on improving plant growth.

## 1. Introduction

Root morphological traits explain a large part of the physiology of roots [1] and the plant’s ability to respond to inputs and environmental constraints [2]. Belowground indicators and allometric relations between and within shoot and root are therefore commented in terms of strategy of plants in dealing with anthropic and natural factors and their ability to provide products and ecosystem services [3,4].

Root traits and allometric relations have therefore been strongly suggested for inclusion in selection criteria for plants (e.g., [5,6] and for the evaluation of agronomic practices [2]). Translating seedling traits into mature plants morphology and behavior, though, is the object of controversy. For wheat many studies report that seedling traits are not related to root architecture at anthesis due to interactions with the environment (e.g., [7,8]). Nevertheless early traits correlate well with vegetative growth, field establishment, exploration of the environment and competition [9] and may relate to later root traits if referred to specific functions such as penetration of hard layers or specific environmental conditions [5,10].

In wheat, root traits have been shown to differ between varieties (e.g., [7,11,12]) and desirable traits are different based on environmental constraints [5]. Plant phenotyping on 36 genotypes chosen along the key steps of evolution in tetraploid wheat from wild emmer to emmer to durum wheat indicates that domestication has changed shoot and root mass and biometric traits [13], although [14] showed that experimental growth conditions such as soil type may affect the extent and direction of the observed changes therefore conclusions are not easy to generalize. It is still considered essential to explore both genetic variation within modern genotypes, and traits of landraces and ancient wheats [3]. Based on root biometrics and allometric relations ancient or underutilized wheat germplasm has been classified in the water-spending plant type [15] since their extended root systems with high length density allows them cope with drought avoidance via rapid uptake of water. This allows to face dry spells without reducing yield, but at the cost of high water consumption, and is considered an efficient strategy in environments where wheat can rely on in-season rainfall with occasional water shortages, as opposed to water-saving wheat varieties which are more successful in environments where wheat grows on stored water [4]. Nevertheless, in spite of the low harvest index of tall ancient phenotypes in high input conditions, some ancient wheats may provide valuable traits in erratic environment and input due to high plasticity in carbon allocation which results in favorable allometry or root traits under drought [3,12]. Research on root and allocation traits of ancient wheats though is still scarce.

Differences in soil-plant interactions between and within plant species are also related to root exudation and relations with rhizosphere organisms. In tetraploid wheat [14] showed that in addition to a strong effect of soil type, genotypic differences were found in the composition of rhizosphere metabolites between domestication groups, as some of the metabolites have high heritability and [16] found that wheat cultivar variability was linked to root traits such as mass, surface area and volume, but also to carbohydrate exudation and diversity of groups of associated rhizospheric organisms.

The rhizosheath is defined as the portion of soil that adheres to the root system of certain plant species [17]. It is a trait emerging from root-soil interactions via the exudation of mucilages which are responsible for key properties of the rhizosheath by binding soil, affecting root hydration, plant water relations and resilience in dry environments [18,19,20], soil stability [20] and relations with soil chemicals [21]. The extent of rhizosheath depends on soil conditions, especially water content [22]. Since it is related to exudates produced by roots and associated microflora, and given its key role in root physiology, the rhizosheath may be considered as a plant-related trait [20]. It is affected by plant species [23,24] and variety [19,25]. In wheat varietal differences have been shown [19] but the production of rhizosheath in ancient wheats has not been investigated yet.

The filamentous ascomycetous *Trichoderma* spp. (family *Hypocreaceae*) are avirulent opportunistic plant symbiont fungi able to colonize soil, plant roots and plant debris. These fungi exhibit different activities for agriculture applications related to their biological functions, such as plant growth promotion, biocontrol of plant disease, organic matter decomposition, bioremediation of pesticides [26].

In particular, *Trichoderma* spp. are known to ameliorate plant performance thanks to their double ability to improve plant growth and development, and to induce plant defense responses against several pathogens, insects and abiotic stress [19,27,28,29,30]. For this reason *Trichoderma*-based products are a major source of registered biofertilizers and biofungicides that can be used in crop sustainable management as biostimulants and for plant disease control [31,32,33]. Seed coating with a commercial product containing *Trichoderma harzianum* T-22 has been shown to improve seedling vigor and also to enhance the protection against the fungal pathogen *Fusarium culmorum* in the susceptible durum wheat (*Triticum durum* L.) cv. Karim [34]. When applied to seeds, the spores of some strains of *Trichoderma* can germinate rapidly, and within few days hyphae and branching filaments grow from the seed onto the emerging radicle [30]. On the other hand, *Trichoderma atroviride* applied as growth medium or seed treatment induced a diverse and distinctively modulated wheat (*Triticum aestivum* L.) root exudate metabolic profile during root colonization [35].

The effects of *Trichoderma*-based biostimulants and/or biopesticides on nutrient uptake, crop growth and systemic resistance have been indicated as species- and genotype-dependent [36,37]. Changes in phytohormone levels, in particular auxins and cytokinins have been established to be one of the direct mechanism by which *T. harzianum* is able to promote tree plant growth and cell wall suberification in the exoderm and endoderm [38,39]. Furthermore, different degrees of *Trichoderma* strain-dependent in root colonization of wheat have been demonstrated due to a diverse production of eight different phytohormones, including gibberellins, abscisic acid (ABA), salicylic acid (SA), auxin IAA (indole-3-acetic acid), and cytokinins [40]. *Trichoderma* spp. has also been shown to increase plant lateral root growth and root hair via IAA (promoting elongation) [41,42] and other mechanisms like plasma membrane H+ATP-ase activation which promotes cell growth [43].

*Trichoderma* spp. are frequently found in agricultural soils as common inhabitants of the rhizosphere because they grow along the entire length of plant root system, colonizing the first or second layer of root epidermis cells [44]. They have also been isolated in the rhizosheath of plants [22], but the ability of *Trichoderma* spp. to affect the amount of rhizosheath soil binding to plants upon inoculation has not been investigated so far.

Among ancient tetraploid wheats Saragolle Lucana was the first italian landrace enrolled in the Italian Wheat Landrace Conservation Registry in 2014 (https://www.gazzettaufficiale.it/eli/gu/2014/01/28/22/sg/pdf, accessed date 1 December 2021). Its early biometrics, root morphology and interactions with plant growth promoting organisms have not been investigated.

We conducted a research on Saragolle Lucana compared with three italian tetraploid wheats corresponding to different times of release and breeding history, inoculated with *Trichoderma harzianum* T-22 (T-22) with the aim of testing the following hypotheses:

(1) that allocation, root traits and rhizosheath soil adhering to roots of Saragolle Lucana are different from those of modern wheats

(2) That T-22 inoculation enhances rhizosheath formation

(3) That effects of T-22 inoculation on plant growth, allocation and early root traits differ between an ancient wheat and modern varieties.

## 2. Results

### 2.1. Overall Growth

**Figure 1:**
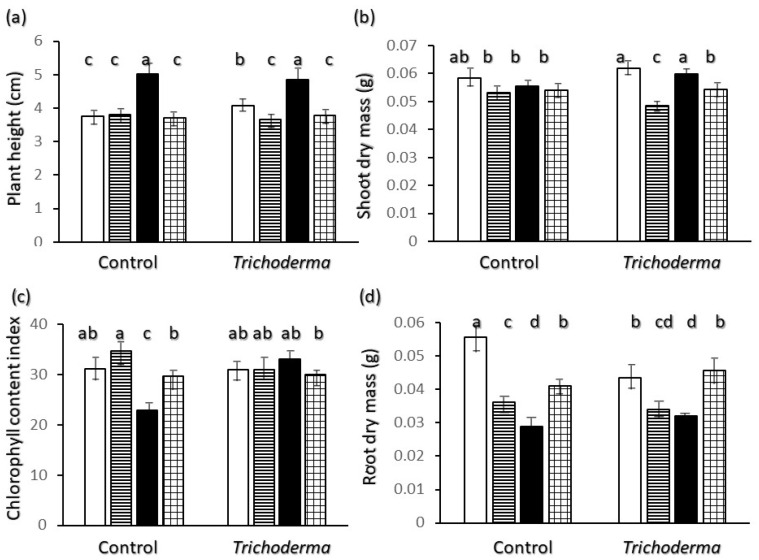
Interaction of wheat variety x *Trichoderma harzianum* T-22 inoculation on overall growth data: (**a**) height to the uppermost node; (**b**) shoot dry mass; (**c**) Chlorophyll Content Index; (**d**) root dry mass. Color code of bars: white = Ciclope; horizontal stripes = Creso; black = Saragolle; grid = Simeto. Vertical segments on each bar indicate standard deviation *n* = 6. Different letters on each bar designate significantly different values for *p* < 0.05 at the Tukey’s post hoc mean separation test.

The height of plants at the uppermost node (Figure 1a) showed significantly highest values for Saragolle and was not significantly different among the other wheat varieties within the control treatment. Values were not significantly affected by *Trichoderma* inoculation except for the Ciclope variety which showed a significant increase, but remained lower than in Saragolle. The same trend was found for height at the tip of the last fully expanded leaf (data not shown). Shoot dry biomass was not significantly different between varieties in the control treatment, but *Trichoderma* caused a significant increase in shoot dry biomass for Saragolle (Figure 1b), whereas it decreased biomass accumulation in Creso. *Trichoderma* inoculation also resulted in a significant increase of the Chlorophyll content index CCI by 45% for Saragolle (Figure 1c) whereas for all varieties non-significant variations were found. The lowest CCI values were recorded for Saragolle control plants (Figure 1c).

Root biomass (Figure 1d) in control plants was significantly lowest for Saragolle and highest for Ciclope, and for the latter variety it was significantly higher in the control than in the *Trichoderma* treatment. Upon inoculation the root mass of Simeto was not significantly different from that of Ciclope. In the control treatment total plant biomass was highest for Ciclope (0.114 g) and lowest for Saragolle (0.084 g), but for the latter variety it increased to 0.092 g upon inoculation with T-22.

### 2.2. Root Biometrics

**Figure 2:**
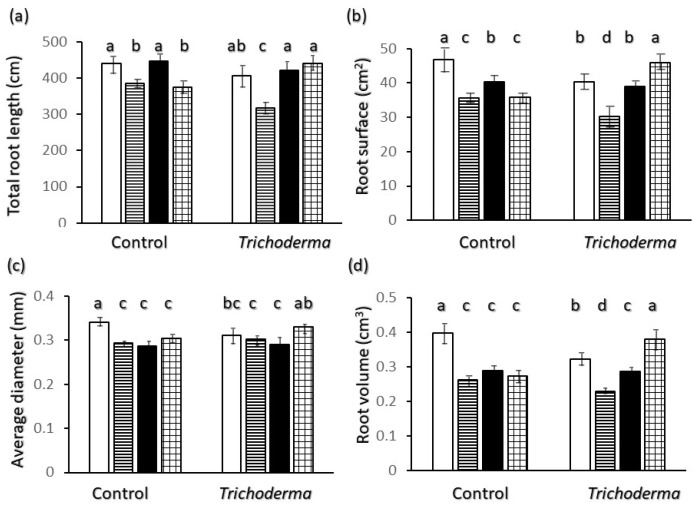
Interaction of wheat variety x *Trichoderma harzianum* T-22 inoculation on root biometrics: (**a**) total length of root structures; (**b**) root surface; (**c**) average diameter; (**d**) root volume. Color code of bars: white = Ciclope; horizontal stripes = Creso; black = Saragolle; grid = Simeto. Vertical segments on each bar indicate standard deviation *n* = 3. Different letters on each bar designate significantly different values for *p* < 0.05 at the Tukey’s post hoc mean separation test.

Non-inoculated plants showed a significantly higher total length of root structures (Figure 2a) in Ciclope and Saragolle compared to other varieties, whereas root surface (Figure 2b) and volume (Figure 2d) were highest in Ciclope, and this corresponds to a thicker root system characterized by a higher average diameter in the latter variety (Figure 2c). *Trichoderma* made roots of Ciclope finer on average as shown by a root diameter decrease by 9.0 % (Figure 2c) and thicker in Simeto with an increase in diameter by 8.7%.

The effect of T-22 was also to increase root length, surface and volume in Simeto (Figure 2a,b,d), whereas for the other varieties such root trait values were reduced or did not show significant differences.

**Figure 3:**
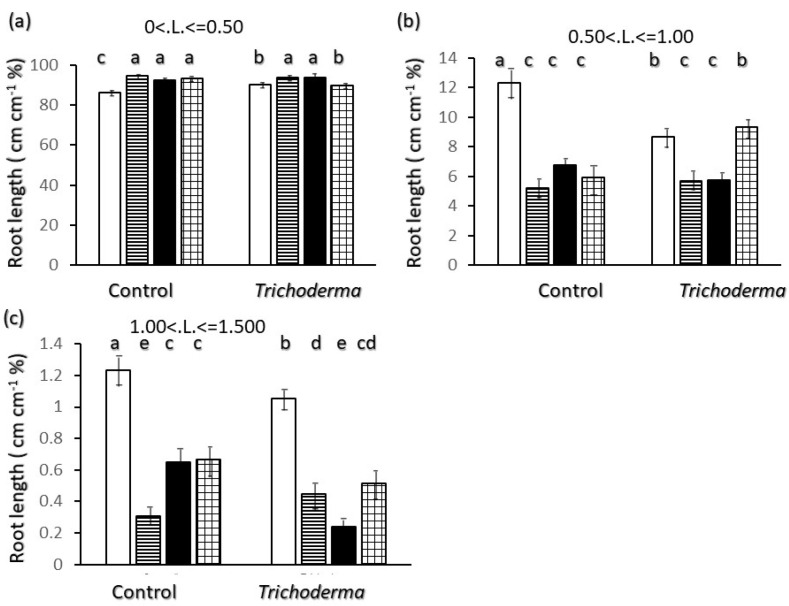
Interaction of wheat variety x *Trichoderma harzianum* T-22 inoculation on root length percent distribution by diameter class: (**a**) diameter class < 0.5 mm; (**b**) diameter class 0.5 < d < 1 mm; (**c**) diameter class 1 < d < 1.55 mm. Color code of bars: white = Ciclope; horizontal stripes = Creso; black = Saragolle; grid = Simeto. Vertical segments on each bar indicate standard deviation *n* = 3. Different letters on each bar designate significantly different values for *p* < 0.05 at the Tukey’s post hoc mean separation test.

Most of the root system length was due to very fine roots (<0.5 mm diameter) (Figure 3a), and in control plants a significantly lower percentage of finest root length was found in Ciclope with around 86% roots in this class than in the other varieties were finest roots represented more than 90% of the total root length. Conversely a higher percentage of roots were found for Ciclope in the diameter classes of 0.5 mm up to 1.5 mm (Figure 3b,c).

At diameters higher than 1.5 mm no significant differences were found due to high variability. *Trichoderma* inoculation significantly increased the proportion of very fine roots for Ciclope (Figure 3a) but values stayed significantly lower than those of Saragolle and Creso. A corresponding reduction in the proportion of roots in the 0.50–1 mm and 1–1.5 mm classes was shown for Ciclope upon inoculation (Figure 3b,c). The only variety showing a decrease in very fine roots (<0.5 mm) and an increase in the 0.50–1 mm class in the *Trichoderma* treatment was Simeto, and this corresponds to an increase in average root diameter upon inoculation for this variety as shown in Figure 2c.

**Figure 4:**
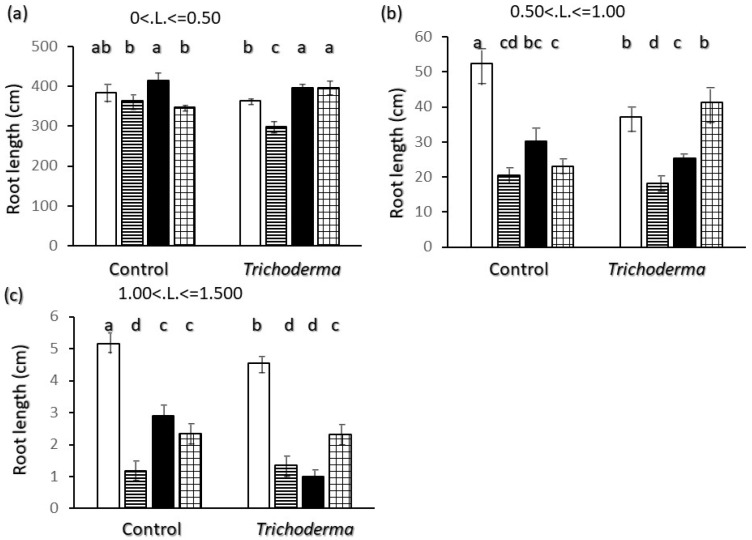
Interaction of wheat variety x *Trichoderma harzianum* T-22 inoculation on root length values by diameter class: (**a**) diameter class < 0.5 mm; (**b**) diameter class 0.5 < d < 1 mm; (**c**) diameter class 1 < d < 1.55 mm. Color code of bars: white = Ciclope; horizontal stripes = Creso; black = Saragolle; grid = Simeto. Vertical segments on each bar indicate standard deviation *n* = 3. Different letters on each bar designate significantly different values for *p* < 0.05 at the Tukey’s post hoc mean separation test.

Length data in absolute values (Figure 4) show that Saragolle had more than 390 cm of total length of very fine roots per plant (Figure 4a), which is the highest value between varieties but not significantly higher than Ciclope in the control and Simeto in the T-22 treatment. Absolute total length corresponding to higher diameter classes up to 1.5 mm (Figure 4b,c), instead, was significantly higher in Ciclope. Simeto is the only variety where the absolute value of root length increased upon inoculation in the <0.5 and 0.5–1 mm diameter classes.

**Figure 5:**
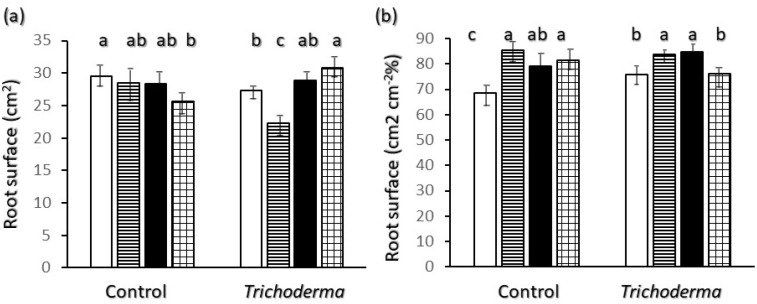
Interaction of wheat variety x *Trichoderma harzianum* T-22 inoculation on root surface in the diameter class < 0.5 mm: (**a**) total length of root structures; (**b**) percent length of root structures. Color code of bars: white = Ciclope; horizontal stripes = Creso; black = Saragolle; grid = Simeto. Vertical segments on each bar indicate standard deviation *n* = 3. Different letters on each bar designate significantly different values for *p* < 0.05 at the Tukey’s post hoc mean separation test.

Figure 5 reports root surface in the finest root class. Control plants showed that absolute values (Figure 5a) of Ciclope were higher than those of Simeto but in percentage of the whole root system (Figure 5b) the former variety showed significantly lower values than the other varieties in this diameter class. Patterns of absolute (Figure 5a) and percent (Figure 5b) values of very fine root surface of plants treated with T-22 are parallel to the corresponding values of root length (Figure 3a and Figure 4a).

### 2.3. Allometric Relations

**Figure 6:**
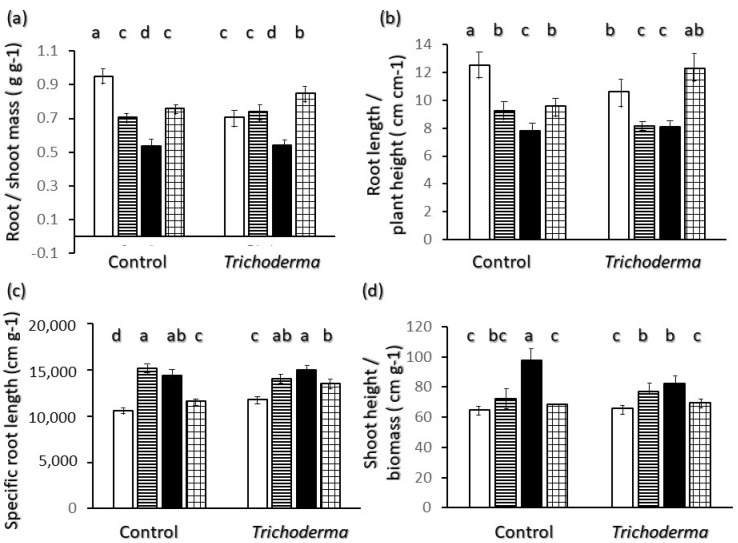
Interaction of wheat variety x *Trichoderma harzianum* T-22 inoculation on Allometric relations of plant parts: (**a**) root to shoot mass ratio *n* = 6; (**b**) root length to plant height ratio Vertical segments on each bar indicate standard deviation *n*=3; (**c**) specific root length *n* = 3; (**d**) shoot height to biomass ratio *n* = 6. Color code of bars: white = Ciclope; horizontal stripes = Creso; black = Saragolle; grid = Simeto. Different letters on each bar designate significantly different values for *p* < 0.05 at the Tukey’s post hoc mean separation test.

Figure 6 reports mass and length relations between different plant traits. The two extremes for root to shoot mass and length ratios (Figure 6a,b) in control plants are Saragolle and Ciclope. Plant root mass (Figure 6a) corresponds to little over 55% of the shoot mass for Saragolle, and almost 95% in Ciclope, and root length (Figure 6b) is almost 8 times the shoot length for Saragolle and almost 13 times for Ciclope. Upon inoculation with *Trichoderma* Ciclope is shown to invest proportionally significantly less in roots in terms of mass (Figure 6b) and length (Figure 6b) whereas Simeto invests proportionally more, but these two varieties still show significantly higher values than Saragolle for the mass ratio (Figure 6a) and than Saragolle and Creso for the length ratio (Figure 6b). The root system’s ratio of length to weight (Figure 6c) shows higher values for Saragolle and Creso, and lowest for Ciclope, corresponding to a thicker root system. The effect of *Trichoderma* was to significantly increase specific root length only in the two varieties characterized by lower values: Ciclope and Simeto, but even so they were still significantly lower than values of Saragolle and Creso. The ratio of shoot height to biomass (Figure 6d) show that values were significantly and considerably higher in Saragolle than in the modern wheats showing a higher proportional investment in height than in biomass for this ancient wheat. Values decreased significantly upon T-22 inoculation for Saragolle only (Figure 6d).

### 2.4. Rhizosheath

**Figure 7:**
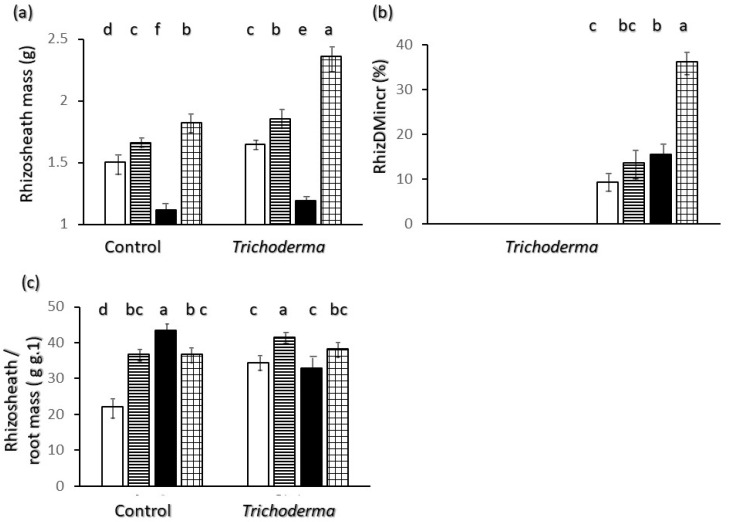
Rhizosheath mass data and indices. Interaction of wheat variety x *Trichoderma harzianum* T-22 inoculation on (**a**) rhizosheath mass; (**c**) rhizosheath/root mass ratio. Main effect of wheat variety on rizosheath percent weight gain upon inoculation RhizDMIncr (**b**). Color code of bars: white = Ciclope; horizontal stripes = Creso; black = Saragolle; grid = Simeto. Vertical segments on each bar indicate standard deviation *n* = 3. Different letters on each bar designate significantly different values for *p* < 0.05 at the Tukey’s post hoc mean separation test.

Figure 7 reports data on rhizosheat amount and relations with root traits. The dry weight of rhizosheath was lowest for Saragolle and highest for Simeto (Figure 7a).

Upon inoculation with T-22 the amount of rhizosheath was higher than in the control for all varieties (Figure 7a). The percent increase (Figure 7b) was lower than 10% in Ciclope and higher than 35% for Simeto. Figure 7c shows that the proportion of rhizosheath to root weight was quite high: the rhizosheath amounted to more than 20 times the weight of roots in Ciclope (the significantly lowest value) and more than 43 times in Saragolle (the significantly highest value) for control plants. Upon inoculation with *Trichoderma* values for Saragolle were significantly lower than in the control while for Ciclope and Creso they were higher or not different, and in general the range of values between varieties was narrower (Figure 7c); in inoculated plants Creso showed a significantly higher value than other varieties. (Figure 7c).

## 3. Discussion

### Shoot and Root Growth and Relations

Data in our experiment show different patterns of biomass allocation and root morphology in the early growth of wheat varieties. The two opposite extremes of overall behavior for non-inoculated plants are the ancient wheat Saragolle and the modern variety Ciclope. Saragolle shows the tallest seedling (Figure 1a) and a shoot growth characterized by a very high proportional investment in height per unit biomass (Figure 6d), as well as the lowest root mass (Figure 1d) and proportional investment in root mass (Figure 6a) and length (Figure 6b). Ciclope shows the highest shoot (Figure 1b) and root (Figure 1d) dry mass and the highest proportional mass and length investment in roots (Figure 6a,b); shoots show the lowest investment in height per unit biomass(Figure 6c).

Patterns of growth and morphology of wheat varieties have been commented in terms of strategy [4]. Also, allocation of assimilates between above- and below-ground plant parts is classically analyzed within the framework of hypotheses derived from the functional balance theory [45], whereby the relative sizes of roots and shoots are an inverse function of their activity. Typical activities for shoot and root within this framework are respectively capture and conversion of light into assimilated carbon, and uptake and transport of nutrients [46] and water. According to this theory a plant exhibiting efficient photosynthesis will have a relatively small shoot compared to a plant with inefficient light capture, which will need a larger shoot mass to provide a unit mass of roots with the assimilates it needs. Along this line the early patterns of mass accumulation and partitioning shown by Saragolle with tall plants and a low root/shoot ratio indicate a competition-oriented strategy, especially for light, and possibly the need to compensate for lower efficiency in resource acquisition. This corresponds to a lower chlorophyll content index of Saragolle (Figure 1c) compared to modern wheats, and points to a less efficient capture and conversion of light. A functional-balance type interpretation is also corroborated by a higher root/shoot ratio exhibited by Saragolle upon T-22 inoculation in coincidence with a significantly higher CCI, therefore a more efficient shoot. Low chlorophyll content values for ancient wheats compared to modern varieties were reported by [3], as in our experiment, and commented in terms of lower yield potential. Conversely a high CCI and investment in belowground biomass allocation for Ciclope suggests a higher shoot efficiency and a strategy oriented towards root growth and soil resource acquisition. This resulted in the highest total biomass produced in our experiment.

Regarding belowground morphology and allometry only, two different root physiognomies emerge: Saragolle and Creso are characterized by a finer root system (lower biomass-Figure 1d, diameter-Figure 2c and higher specific root length-Figure 6c) whereas the more recent Ciclope and Simeto exhibit a thick system with high biomass and diameter and lower specific root length, and show a high presence of roots larger than 0.5 mm diameter in control plants for Ciclope and inoculated plants for Simeto. The high mass investment and thickness-though-allows a high surface and volume. In spite of the lowest mass investment in roots, thanks to a finer root system Saragolle has absolute values of total (Figure 2a) and fine (Figure 4a) root length as high as Ciclope and a higher percentage of fine roots like Creso and Simeto (Figure 3a). This allows a comparable absolute surface but a higher percent surface than Ciclope (Figure 5). This adds to aboveground patterns in drawing the strategy of Saragolle as strongly oriented towards the occupation of space by maximizing the length of shoots and root per unit biomass. No data on the root system of Saragolle are available in the literature for comparison but the root profile of Saragolle emerging from our results corresponds to the profile set by [3,11] for ancient wheats including underutilized species: a high root length and specific root length with investment in lengths and surfaces rather than mass. Authors emphasize how this type of plants achieve a more extensive root system, resulting in higher soil volume exploration and acquisition capacities for below-ground resources compared to modern wheats, and this is achieved by using fewer assimilates to build up root length, surface and volume.

Differences between ancient wheats and modern dwarf lines were investigated by [47] who found a higher root length in modern lines from gel chamber experiments but a lower length from field experiments. They also report that in spite of differences in length, root diameter was not altered by dwarfing genes and hypothesized a direct effect of dwarfing alleles on root growth during seedling establishment, rather than a secondary partitioning effect. Root systems of high length or length density have been identified as successful in limiting drought damage to yield, but there is no consensus as to whether root length in surface [7] or deep [5] layers is more desirable, and this is also linked to weather and water supply patterns [11,48]. Root mass seems more correlated with nutrient uptake than with water relations [49]. A large percentage of fine roots in ancient wheats is discussed in terms of advantage in fast water uptake during drought (water spenders) but not water use efficiency [11].

The variation in rhizosheath mass between varieties found in our experiment is comparable or lower than that reported in the literature. For wheat a three-fold variation of rhizosheath to root mass between varieties has been reported [19] whereas in our case the minimum value found in control Ciclope was about half that of control Saragolle which showed the maximum value of relative rhizosheath mass (Figure 7c). This is due to the much lower root mass of Saragolle compared to Ciclope which more than compensates the lowest absolute value of rhizosheath mass (Figure 7a). Finding Ciclope and Saragolle at the opposite extremes for relative rhizosheath mass data confirms what discussed for the opposite profiles of such two varieties regarding shoot and root growth. Simeto was the variety with highest absolute values of rhizosheath and percent increase of rhizosheath mass upon inoculation with T-22. Rhizosheath results add to growth and morphology in showing that Simeto had a distinctive profile where a rather large and thick root system is accompanied by a higher response to inoculation and a large amount of rhizosheath. This is somewhat contrary to literature reports where a larger rhizosheath is found in finer root systems (e.g., [23]). Also a comparison of ancient and modern wheats inoculated with Plant Growth Promoting Rhizobacteria (PGPR) suggests a reduced ability of modern cultivars to interact with PGPR [50]. The higher absolute and percent response of Simeto to inoculation with a fungal product based on *Trichoderma harzianum* T-22 in our data does not confirm a lower ability of modern wheats to entertain relations and respond to rhizosphere interactions.

Rhizosheaths have been mainly reported in terms of adaptation to arid environments [20] by enhancing water retention and preventing root dehydration whereas the role in water uptake is controversial [18]. Wheat varieties with larger rhizosheath have been shown to sustain higher transpiration in dry condition and stay alive beyond the permanent wilting point of varieties with smaller rhizosheath [19]. Alterations in soil porosity due to mucilages involved in rhizosheath formation [20] may play a role in continuity of water movement in conditions of alternating water supply. Rhizosheats also play a role in plant nutrition and soil penetration (e.g., [51]) therefore species and varietal differences in rhizosheath may be viewed as a generally advantageous trait, especially in conditions of low input and alternating conditions.

All wheat varieties showed a significantly higher rhizosheat mass in response to *Trichoderma* inoculation and this allows us to reject the null hypothesys with respect to the effect of T-22 on rhizosheath enhancement. To the best of our knowledge this is the first report of such an effect and it opens the way to new interpretations of effects of *Trichoderma* inoculation on improving plant growth: besides documented mechanisms based on growth hormones and resistance to pathogens (e.g., [30,39]), *Trichoderma* treatments may promote a better performance of plants through rhizosheath-mediated improvements of water relations [18], nutrition [51] and soil structure [20,21].

## 4. Materials and Methods

The experimental design was a factorial combination of (A) four tetraploid wheats (*Triticum durum* Desf., 2n = 4x = 28; AABB genome) and (B) two inoculation treatments with six replications.

### 4.1. Tetraploid Wheats

Italian tetraploid wheats consisted of three modern hard wheat varieties released in different years and an ancient wheat landrace:

Creso (1974, Yaktana-54/Norin 10-B//2*Cappelli-63/3/3*Tehuacan-60/4/Capelli-B144);

Simeto (1988, Capeiti-8/Valnova)

Creso and Simeto varieties were obtained by mutagenesis and crosses involving old wheat materials;

Ciclope (Trinakria/Berillo//Valnova/Trinakria) variety released in 2006, selected from crosses between breeding lines [52,53];

Saragolle Lucana: the first Italian landrace enrolled in the Italian Wheat Landrace Conservation Registry in 2014 (https://www.gazzettaufficiale.it/eli/gu/2014/01/28/22/sg/pdf, accessed date: 1 December 2021). It is an ancient durum wheat population safeguarded from the risk of genetic erosion through in situ/on farm conservation and cultivation in the northern Basilicata (Sud Italy) area by the “Associazione Lucana Cerealisti di Antiche Varietà, ALCAV”.

All four tetraploid wheats are referred to as varieties in the text.

### 4.2. Inoculation Treatments

Inoculation treatments were:

(i) Seed coating with a spores suspension of the commercial formulation Trianum P (Koppert, Berkel en Rodenrijs, The Netherlands) containing *Trichoderma harzianum* Rifai KRL-AG2 (T-22);

(ii) Seed coating with water (control without T-22).

### 4.3. Seedling Growth Conditions

Seeds were selected for weight uniformity within variety (Saragolle Lucana 68.5 ± 0.46 mg; Creso 48.6 ± 0.32 mg; Simeto 50.6 ± 0.33 mg; Ciclope 64.6 ± 0.41 mg). They were surface-sterilized with 0.6% Na-hypochlorite solution for 2 min, then with 70% ethanol for 2 min and then rinsed three times with sterile dH_2_O.

Seeds for the *Trichoderma* treatment were coated with a mixture of a T-22 suspension at concentration of 10^6^ spores mL^−1^, or water as control, in 4 μL seed^−1^ of Tween-20, applied to seeds guaranteeing a homogeneous distribution by continuous rotation, until complete adhesion and absorption, according to [34,54].

Two seeds were sown per pot, irrigated and thinned to 1 seedling per pot after 9 days. Pots were cylinders of 25 cm length and 2.8 cm diameter and were filled with 172.0 g +/− of a field collected silty loam soil with the following characteristics: sand (50–2000 μm) 43.6%, silt (2–50 μm) 34.2%, clay < 2 μm) 22.1%., pH 6.8; N 1.9 g kg^–1^; phosphates (P_2_O_5_) 50.3 g kg^–1^; potassium oxide (K_2_O) 1430 g kg^–1^.

Pots were arranged randomly in a custom-built growth cabinet and grown for 20 days after emergence (average T = 21.5 °C; average relative humidity 33.6%). After filling pots were brought to field capacity and thereafter irrigated every 3 days to replace 100% of gravimetrically-determined evapotranspiration.

### 4.4. Measurements

At the end of the experiment the following measurements were made:

Chlorophyll content index (CCI) on three leaves per plant with a leaf transmittance leaf clip chlorophyll concentration meter (MC-100 Apogee instruments U.S.A.):

CCI = T_931_/T_653_

where T_931_ = Leaf transmittance at 931 nm; T_653_ = Leaf transmittance at 653 nm

Biometric measurements: plant height to the uppermost internode and to the tip of the last fully expanded leaf, above-ground plant fresh and dry (after oven drying at 70 °C until constant weight) biomass after clipping at the soil level. The bottom of pots was removed and the soil was gently pushed from the bottom. On three replications the root system was extracted by washing over a mesh of 0.5 mm and placed in a transparent tray (200 × 250 mm) with a 4-mm to 5-mm deep layer of water and scanned by STD 4800 Image Acquisition System at 1200 DPI. After scanning roots were blotted and weighed to obtain the fresh root mass, then oven dried at 70 °C until constant weight to obtain the root dry mass. Root morphology was determined on scanned images using WinRhizo.

ArabidopsisV2009c image analysis software (Regent Instruments Inc., QC, Canada). The following traits were measured: total length (cm), surface area (cm^2^), mean diameter (mm), volume (cm^3^). All parameters were assessed on a plant basis, and the distribution of length, surface and volume were then classified into seven diameter classes from 0.0 to 3.5 mm in 0.5 mm increments. No roots with diameter larger than 3.5 mm were found.

We then calculated root to shoot biomass ratio (g g^−1^), root length to plant height ratio (cm cm^−1^), specific root length (root length per unit of root biomass (cm g^−1^) and shoot height to mass ratio (cm g^−1^).

On three replications the mass of rhizosheat soil was determined as follows: the root system was held by the plant basis and gently shaken free of bulk soil. The root was then transferred to a clean sheet of paper and the soil which was not firmly attached to roots was gently brushed off with a soft brush. The soil which remained attached to roots after this treatment was considered rhizosheath soil. The rhizosheath-root complex was then weighed to determine the fresh mass (RRhizFM g plant^−1^), oven-dried at 105 °C and weighed again to obtain the dry mass (RRhizDM g plant^−1^). Roots were thereafter washed over a 0.5 mm mesh, blotted and weighed to obtain the fresh root mass (RFM g plant^−1^), then oven dried at 70 °C until constant weight to obtain the root dry mass (RDM g plant^−1^). The rhizosheath soil fresh (RhizFM) and dry (RhizDM) mass were then calculated as:

RhizFM = RRhizFM-RFM g plant^−1^

and

RhizDM = RRhizDM-RDM g plant^−1^

We then calculated the percent increase of RhizDM following inoculation (RhizDMincr) as:

RhizDMincr = 100 * (RhizDM of inoculated plants-RhizDM of control plants)/RhizDm of control plants.

As a consequence of the described procedures we had 6 replicated for the following variables: plant height, shoot dry mass, chlorophyll content index, root dry mass, and derived indices; the number of replicates was 3 for root length, surface area, diameter, volume, RhizFM, RhizDM, RhizDMincr and derived indices.

One-Way ANOVA was performed to test the significance of the main factor (A) wheat variety on RhizDMinc. Two-way ANOVA was performed to test the significance of the factorial combination of the two main factors (A) wheat variety and (B) inoculation treatments and their interaction for all other traits. Mean separation was performed using the post-hoc test of Tukey at *p* 0.05.

## Data Availability

Data presented in this study are available on request from the corresponding author.
